# Performance Limits of GNSS Code-Based Precise Positioning: GPS, Galileo & Meta-Signals

**DOI:** 10.3390/s20082196

**Published:** 2020-04-13

**Authors:** Priyanka Das, Lorenzo Ortega, Jordi Vilà-Valls, François Vincent, Eric Chaumette, Loïc Davain

**Affiliations:** 1 Institut Supérieur de l’Aéronautique et de l’Espace (ISAE-SUPAERO), University of Toulouse, 31055 Toulouse, France; priyanka.das@isae-supaero.fr (P.D.); francois.vincent@isae-supaero.fr (F.V.); eric.chaumette@isae-supaero.fr (E.C.); 2 Safran/Sagem DS, 95610 Eragny, France; 3 Telecommunications for Space and Aeronautics Lab (TéSA), 31500 Toulouse, France; loic.davain@safrangroup.com

**Keywords:** GNSS, Cramér–Rao bound, time-delay estimation, maximum likelihood estimation, code-based positioning, precise positioning, GPS/Galileo signals, Galileo meta-signals

## Abstract

This contribution analyzes the fundamental performance limits of traditional two-step Global Navigation Satellite System (GNSS) receiver architectures, which are directly linked to the achievable time-delay estimation performance. In turn, this is related to the GNSS baseband signal resolution, i.e., bandwidth, modulation, autocorrelation function, and the receiver sampling rate. To provide a comprehensive analysis of standard point positioning techniques, we consider the different GPS and Galileo signals available, as well as the signal combinations arising in the so-called GNSS meta-signal paradigm. The goal is to determine: (i) the ultimate achievable performance of GNSS code-based positioning systems; and (ii) whether we can obtain a GNSS code-only precise positioning solution and under which conditions. In this article, we provide clear answers to such fundamental questions, leveraging on the analysis of the Cramér–Rao bound (CRB) and the corresponding Maximum Likelihood Estimator (MLE). To determine such performance limits, we assume no external ionospheric, tropospheric, orbital, clock, or multipath-induced errors. The time-delay CRB and the corresponding MLE are obtained for the GPS L1 C/A, L1C, and L5 signals; the Galileo E1 OS, E6B, E5b-I, and E5 signals; and the Galileo E5b-E6 and E5a-E6 meta-signals. The results show that AltBOC-type signals (Galileo E5 and meta-signals) can be used for code-based precise positioning, being a promising real-time alternative to carrier phase-based techniques.

## 1. Introduction

Synchronization is a key first stage in many applications, e.g., radar, sonar, communications, or navigation, to name a few [1,2,3,4,5]. This typically implies the estimation of both propagation delay and Doppler shift (or higher order Doppler terms in high dynamics scenarios), in order to identify, localize, and/or track radiating sources. Several approaches and estimators are available in the literature to perform such synchronization, for instance, standard Global Navigation Satellite Systems (GNSS) receiver architectures rely on a scalar (i.e., different satellite signals are processed with independent channels) acquisition and tracking approach. In this case, the former provides a coarse point estimate of the synchronization parameters (identify/acquire visible satellites), and the latter keeps track of their time-varying evolution [6]. These two stages, i.e., acquisition and tracking, can be seen as particular instances of the Maximum Likelihood (ML) solution. The optimal positioning solution is the so-called Direct Position Estimation (DPE) [7,8], that is, the direct ML position estimation from the sum of signals to all visible satellites. Indeed, the set of time-delays and Doppler shifts to each individual satellite are related to the same receiver position; thus, not exploiting them together is suboptimal because we are not taking into account the geometry constraint. However, DPE is not useful in practice because it requires solving a high-dimensional optimization problem. Therefore, thanks to the quasi-orthogonality of GNSS signals, the standard solution is a two-step (synchronization + multilateration) positioning approach: (i) first, individual time-delay estimates to each visible satellite are used to build a set of independent pseudoranges; and (ii) then these pseudoranges are used used to solve a multilateration problem to estimate the user position, typically via a weighted least-squares (WLS) [9]. Even if DPE approaches are known to provide better position estimates under certain conditions, it has been recently shown that the traditional two-step approach is asymptotically optimal [10]. Then, the ultimate achievable standard point positioning (SPP) GNSS receiver performance is directly driven by the achievable time-delay estimation performance. By ultimate performance, we refer to the asymptotic region of the time-delay ML estimator. In that perspective, we do not assume external errors such as ionospheric/tropospheric delays, orbital or satellite clock errors, or environment-specific effects such as multipath [9]. For a comprehensive analysis on the specific impact of such external errors and different types of corrections, refer to [11]. The asymptotic region is characterized considering only the thermal noise and a high signal-to-noise (SNR) ratio to ensure the efficient behavior of the ML estimator. Moreover, these external errors are the same whatever the processing, so that we do not need to consider them with an aim of comparing SPP and Precise Point Positioning (PPP) schemes.

Notice that the standard way to obtain precise positioning solutions is to exploit the signal phase information. Indeed, this measurement is linked to the wavelength which is much smaller than the baseband signal resolution (i.e., for a legacy Global Positioning System (GPS) L1 C/A signal, the wavelength is approximately 19 cm while the baseband signal resolution is 300 m). Unfortunately, exploiting this phase information implies solving a much more complicated problem, mainly because the carrier phase measurement is ambiguous (i.e., unknown number of cycles inside the baseband signal resolution), rather than such ambiguity resolution being the bottleneck [9] (Chapters 21 and 23). Two main approaches are available in the literature: (i) differential techniques such as Real-Time Kinematics (RTK) [9] (Chapter 26); and (ii) PPP techniques [9] (Chapter 25). RTK requires the use of a reference station with a communication link between the two receivers, and is only valid for short ranges from the base-station to ensure that the two receivers observe the same propagation errors. PPP techniques allow us to get rid of the reference station but to reach decimetric precision in turn need: (i) precise carrier phase measurements, which is not the case in harsh propagation conditions; (ii) high accuracy satellite orbits, clock, and propagation (ionospheric and tropospheric) error corrections; and/or (iii) multi-frequency/multi-system architectures to compensate propagation effects. The price to be paid is the need to access a network broadcasting precise corrections (i.e., International GNSS Service (IGS) products), and a long convergence time of tens of minutes. As stated in [12], these drawbacks limit the use of PPP for real-time applications. Then, the fundamental question that ignited this work is: *Can we obtain a GNSS code-only precise positioning solution and under which conditions?*

GNSS positioning being mainly an estimation problem, the optimal performance is given by the well-known Cramér–Rao bound (CRB) [13], which provides an accurate lower bound on the mean square error (MSE) sense under certain conditions (for instance, in the high SNR regime of the conditional signal model [14,15], of interest in this contribution because it is linked to the optimality of the two-step solution). In the GNSS context, this bound is particularly interesting because the synchronization process is based on the ML principle under Gaussian noise assumptions, which is known to reach the CRB (the ML estimator in this case is asymptotically efficient) [15]. In a recent contribution [16], we derived a new compact closed-form CRB for the time-delay estimation of a generic band-limited signal, which is directly computed from the signal samples and perfectly fits the GNSS problem. In addition, in this contribution, we provide a new joint time-delay and phase estimation CRB to support the discussion. In this article we exploit the time-delay CRB expression to compare the achievable synchronization performance with different GPS and Galileo signals, namely: the GPS L1 C/A, L1C, and L5 signals; the Galileo E1 OS, E6B, E5b-I, and E5 signals; and the Galileo E5b/E6 and E5a/E6 meta-signals. Such comprehensive performance analysis from an optimal estimation point of view is not available in the literature, being an important missing point. In addition, the analysis also provides which is the performance threshold that implies a need of carrier phase-based positioning.

The article is organized as follows. Section 2 presents the basic GNSS signal model. Section 3 introduces the time-delay CRB for band-limited signals along with the MLE and a new joint time-delay and phase estimation CRB. Section 4 presents an overview of the GPS civil signals. A complete overview of the Galileo signals is provided in Section 5. The concept of GNSS meta-signals together with the signal structure for the most representative cases is given in Section 6. Section 7 summarizes the main results. Conclusion are drawn in Section 8.

## 2. Signal Model

**Figure 1:**
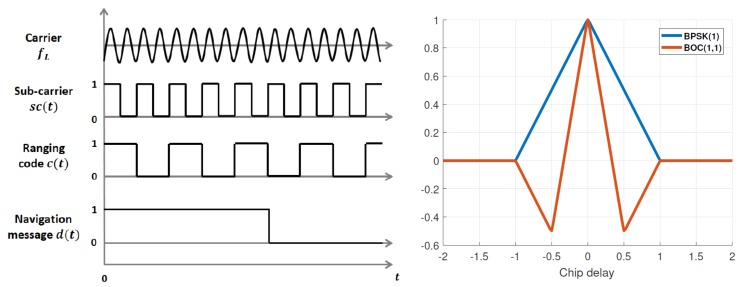
(**Left**) Multilayer GNSS signal structure. (**Right**) ACF for BPSK(1) and BOC(1,1) subcarriers.

We consider the transmission of a band-limited GNSS signal c(t) (bandwidth *B*), so-called Pseudo-Random Noise (PRN) code in the GNSS terminology, over a carrier frequency $f_{c}$ ($\lambda_{c}=\frac{c}{f_{c}}$), from a transmitter (satellite) T to a receiver R. During the observation time, both transmitter and receiver are static, that is, their respective positions is constant $p_{T}\left(t\right)=p_{T}$ and $p_{R}\left(t\right)=p_{R}$. The complex analytic signal at the output of the receiver’s antenna can be written as $x_{A}\left(t\right)=\alpha_{R}c_{R}\left(t\right)+n_{A}\left(t\right)$, with $n_{A}\left(t\right)$ a zero-mean white complex Gaussian noise, and where the gain $\alpha_{R}$ depends on the transmitted signal power, the transmitter/receiver antenna gains and polarization vectors, and the radial distance between T and R, $p_{TR}$ [17,18]. In that perspective, the propagation delay $\tau \left(t\right)$ is constant $\tau \left(t\right)=\tau =\frac{∥p_{TR}∥}{c}$ and the baseband output of the receiver’s Hilbert filter is $x\left(t\right)=\alpha c\left(t-\tau \right)+n\left(t\right)$, with n(t) a complex white Gaussian noise within the filter bandwidth with unknown variance $\sigma_{n}^{2}$, and $\alpha =\alpha_{R}e^{-j2\pi f_{c}\tau }$. The discrete vector signal model is build from $N=N_{2}-N_{1}+1$ samples at $T_{s}=\frac{1}{F_{s}}$,

(1) $$\begin{array}{cc} x & =\alpha c\left(\tau \right)+n, \end{array}$$

where $n∼\mathrm{CN}\left(0,\sigma_{n}^{2}I_{N}\right)$ and $x={\left(x\left(N_{1}T_{s}\right),\cdots ,x\left(N_{2}T_{s}\right)\right)}^{⊤}$, $n={\left(n\left(N_{1}T_{s}\right),\cdots ,n\left(N_{2}T_{s}\right)\right)}^{⊤}$, $c\left(\tau \right)={\left(c\left(N_{1}T_{s}-\tau \right),\cdots ,c\left(N_{2}T_{s}-\tau \right)\right)}^{⊤}$. Since the transmitter/receiver antenna gains and polarization vectors are in general unknown, α is assumed to be an unknown complex parameter as well [1,2,3,18,19]. Thus, the unknown deterministic parameters [20] can be gathered in vector $\underline{\epsilon }={\left(\sigma_{n}^{2},\tau ,\alpha ,\alpha^{*}\right)}^{⊤}$, where $\alpha^{*}$ is the complex conjugate of α. Notice that c(t) can be directly a PRN code with a Binary Phase Shift Keying (BPSK) modulation where there is no subcarrier, as in the case of GPS L1 C/A, or a subcarrier modulated PRN, i.e., using a Binary Offset Carrier (BOC) [21] type modulation. The subcarrier has a direct impact on the correlation function, therefore on the estimation performance. On top of that, the signal may have data bits or not, depending on whether it belongs to a data component or a pilot component. A generic multilayer GNSS signal structure is shown in Figure 1 (left) and the autocorrelation function (ACF) for a standard BPSK(1) and BOC(1,1) in Figure 1 (right). Notice that the correlation peak is narrower for the BOC(1,1), which will in turn lead to a better time-delay estimation. Notice that the model in Equation (1) can be reparameterized as

(2) $$x=\rho c^{\prime }\left(\theta \right)+n,\;c^{\prime }\left(\theta \right)=c\left(\tau \right)e^{j\varphi },\;\rho \in R^{+},\theta^{⊤}=\left(\varphi ,\tau \right),$$

and then the unknown deterministic parameters are $\underline{\epsilon }={\left(\sigma_{n}^{2},\rho ,\theta^{⊤}\right)}^{⊤}$, which are used to derive the new joint time-delay and phase estimation CRB.

## 3. Time-Delay ML Estimation (MLE) and Cramér–Rao Bound

Considering the signal model in Equation (1), the time-delay MLE is defined as (i.e., let $S=span\left(A\right)$, with A a matrix, be the linear span of the set of its column vectors, $S^{⊥}$ the orthogonal complement of the subspace *S*, $\Pi_{A}=A\left(A^{H}A\right)A^{H}$ the orthogonal projection over *S*, and $\Pi_{A}^{⊥}=I-\Pi_{A}$.) [19]

(3) $$\begin{array}{cc} \; & \hat{\tau }=\arg\min_{\tau }\left\{x^{H}\Pi_{c\left(\tau \right)}^{⊥}x\right\}=\arg\max_{\tau }\left\{\frac{{\left|c{\left(\tau \right)}^{H}x\right|}^{2}}{c{\left(\tau \right)}^{H}c\left(\tau \right)}\right\}, \end{array}$$

which is useful to determine the value of ${\mathrm{SNR}}_{\mathrm{out}}$ (threshold), which allows reaching the CRB, because it is known that such estimator is asymptotically efficient (e.g., in the high SNR regime) for the conditional signal model of interest [14,15].

In a recent contribution [16], we derived a new compact closed-form CRB for the time-delay estimation of a generic band-limited signal which only depends on the signal samples, given by the following Fisher Information (FI),

(4) $$\begin{array}{cc} F_{\tau |\underline{\epsilon }}\left(\underline{\epsilon }\right) & =2{\mathrm{SNR}}_{\mathrm{out}}F_{s}^{2}\left(\frac{c^{H}\mathrm{Vc}}{c^{H}c}-{\left|\frac{c^{H}\Lambda c}{c^{H}c}\right|}^{2}\right)\underset{\mathrm{real}\;\mathrm{signal}}{=}2{\mathrm{SNR}}_{\mathrm{out}}F_{s}^{2}\left(\frac{c^{T}\mathrm{Vc}}{c^{T}c}\right), \end{array}$$

where ${\mathrm{SNR}}_{\mathrm{out}}=\frac{{\left|\alpha \right|}^{2}E}{\left(\sigma_{n}^{2}/F_{s}\right)}=\frac{{\left|\alpha \right|}^{2}}{\sigma_{n}^{2}}c^{H}c$ and E is the energy of the signal. Λ and V are defined as (for $N_{1}\leq n,n^{\prime }\leq N_{2}$)

(5) $$\begin{array}{cc} \; & {\left(V\right)}_{n,n^{\prime }}=\left|\begin{array}{c} n^{\prime }\neq n:{\left(-1\right)}^{\left|n-n^{\prime }\right|}\frac{2}{{\left(n-n^{\prime }\right)}^{2}}\\ n^{\prime }=n:\frac{\pi^{2}}{3} \end{array}\right.\;;\;{\left(\Lambda \right)}_{n,n^{\prime }}=\left|\begin{array}{c} n^{\prime }\neq n:\frac{{\left(-1\right)}^{\left|n-n^{\prime }\right|}}{\left(n-n^{\prime }\right)}\\ n^{\prime }=n:0 \end{array}\right.. \end{array}$$

From the FI, the time-delay CRB is written as

(6) $$\begin{array}{cc} \; & {\mathrm{CRB}}_{\tau |\underline{\epsilon }}\left({\underline{\epsilon }}^{0}\right)={\left(F_{\tau |\underline{\epsilon }}\left({\underline{\epsilon }}^{0}\right)\right)}^{-1}, \end{array}$$

with ${\underline{\epsilon }}^{0}$ a selected value of $\underline{\epsilon }$. We can further extend this CRB result to the joint time-delay and phase $\theta^{⊤}=\left(\varphi ,\tau \right)$ estimation resorting to the model in Equation (2), which is useful for the discussion in Section 7.5. The complete derivation of this new CRB expressed from the signal samples is given in Appendix A,

(7) $$\begin{array}{ccc} {\mathrm{CRB}}_{\theta |\underline{\epsilon }} & = & \left[\begin{array}{cc} {\mathrm{CRB}}_{\varphi |\underline{\epsilon }} & {\mathrm{CRB}}_{\tau ,\varphi |\underline{\epsilon }}\\ {\mathrm{CRB}}_{\tau ,\varphi |\underline{\epsilon }} & {\mathrm{CRB}}_{\tau |\underline{\epsilon }} \end{array}\right], \end{array}$$

(8) CRBτ∣ϵ_=12SNRout1Fs2cHVccHc−ImcHΛccHc2=realsignal12SNRout1Fs2cHVccHc,

(9) CRBφ∣ϵ_=12SNRout1+ImcHΛccHc2cHVccHc−ImcHΛccHc2=realsignal12SNRout,

(10) CRBτ,φ∣ϵ_=12SNRoutImcHΛccTcFscHVccHc−ImcHΛccHc2=realsignal0.

Then, we can see that, for a real signal, there is no impact on the time-delay estimation if we consider or not the signal phase, i.e., ${\mathrm{CRB}}_{\tau ,\varphi |\underline{\epsilon }}=0$, the time-delay CRB is the same as the one derived from Equation (4), and the performance on the phase estimation does not depend on the signal, ${\mathrm{CRB}}_{\varphi |\underline{\epsilon }}=\frac{1}{2{\mathrm{SNR}}_{\mathrm{out}}}$.

## 4. GPS Signals

Nowadays, most of the GNSS applications are based on the signals transmitted at the L1 frequency band. Four GPS signals are transmitted at this L1 band: the C/A signal, the precise P(Y) signal, the modern military M signal, and the new L1C civil signal. In addition, other GNSS signals of interest are broadcasted at lower bands, namely GPS L5 and GPS L2 signals. In this section, we provide a brief overview of the GPS civil signals allocated in the L1 and L5 bands.

- *GPS L1 C/A Signal*: The GPS L1 coarse/acquisition (C/A) signal is composed of three components: (i) the NAV message d(t) [22] with a data rate of 50 bits per second. Each bit lasts 20 ms; (ii) the PRN sequence of 1023 chips (i.e., from the Gold codes family [23]), repeated every 1 ms, meaning the system operates with a frequency rate of 1.023 Mchips/s; and (iii) the carrier frequency 1575.42 MHz is used to allocate the BPSK modulation to transmit the GPS L1 C/A signal in the L1 band (i.e., denoted BPSK(1)).
- *GPS L1C Signal*: GPS L1C is a modernized GPS civil signal designed to improve the performance of the legacy GPS L1 C/A signal. As for the Galileo E1 Open Service Signal [24], the Multiplexed BOC (MBOC) modulation is considered, namely a MBOC(6,1,1/11) [25] modulation was selected to transmit the GPS L1C signal. The MBOC(6,1,1/11) is defined in the PSD domain as a mix of a BOC(1,1) and BOC(6,1) modulations, where $\frac{10}{11}$th of the power is associated to the BOC(1,1) modulation and $\frac{1}{11}$th of the power is associated to the BOC(6,1). Note that the BOC modulation is generally denoted BOC(p,q), where *p* refers to the sub-carrier frequency $f_{sc}=p\times 1.023$ MHz (i.e., $sc\left(t\right)=sign\left[\sin\left(2\pi f_{sc}t\right)\right]$ with sign the sign function) and *q* to the ranging code frequency $f_{c}=q\cdot 1.023$ MHz [26]. Then, the PSD of the MBOC modulation can be expressed as,
  (11) $$G_{MBOC(6,1,1/11)}\left(f\right)=\frac{10}{11}G_{BOC(1,1)}\left(f\right)+\frac{1}{11}G_{BOC(6,1)}\left(f\right),$$
  where $G_{BOC(1,1)}\left(f\right)$ is the PSD of a low frequency BOC component and $G_{BOC(6,1)}\left(f\right)$ is the PSD of a high frequency BOC component. This signal can be generated with two different architectures: (i) the Composite BOC (CBOC) modulation [24], which is used in the Galileo E1 open service signal; and (ii) the implementation used for the GPS L1C signal, i.e., a Time-Multiplexing BOC (TMBOC) modulation [25] used to transmit the pilot component of the GPS L1C signal and a BOC(1,1) modulation to transmit the corresponding data component. Notice that the TMBOC modulation divides temporally the power of the BOC(1,1) and BOC(6,1) of the pilot component. Indeed, the pilot component time-series comprises 29/33 BOC(1,1) spreading symbols and 4/33 BOC(6,1) spreading symbols. Moreover, an unequal power distribution is used to transmit the data and pilot components of the GPS L1C signal, i.e., the data and pilot channels represent 25% and 75% of the total power, respectively. The L1C PRN sequences last 10 ms and the CNAV-2 message [27] is transmitted at 100 symbols per second.
- *GPS L5 Signal*: A third GPS civil signal was proposed at the L5 frequency band (1176.45 MHz) [28], in order to support safety-of-life applications. This GPS L5 signal has a clock rate of 10.23 MHz that is 10 times the clock rate of the legacy GPS L1 C/A signal. Moreover, this signal has two different components: (i) a data component (in-phase) used to broadcast the CNAV L5 navigation message [29]; and (ii) a pilot component (quadrature). The GPS L5 PRN sequence has a length of 10230 chips and the the chip rate is the 10.23 Mchips/s, yielding to a PRN sequence period of 1 ms. Each chip is mapped into a BPSK(10) modulation. The L5 CNAV navigation message is transmitted at 100 symbols per second.

## 5. Galileo Signals

Within the Galileo framework, two signals are transmitted in the E1 band. The Public Regulated Service (PRS) signal uses a high-order BOC(15,2.5) subcarrier and the E1 Open Service (OS). The former is confidential, thus it is out of scope of this paper. In addition, other GNSS signals of interest are broadcasted at lower bands, namely Galileo E6 and Galileo E5 signals.

- *Galileo E1 OS Signal*: The Galileo E1 OS signal is constructed as the combination of two CBOC modulations [30] (Chapter 4), a particular implementation of the MBOC modulation. This signal is separated into two components: the data component which transmits the navigation message, called I/NAV message [24], and the pilot component. The I/NAV navigation message [24] is broadcasted at 250 symbols per second, the data PRN sequence of 4092 chips lasts 4 ms and the pilot PRN (combination with a secondary code of length 25) lasts 100 ms. The subcarriers BOC(1,1) and BOC(6,1) in the data component are added in phase (+). On the other hand, the subcarriers BOC(1,1) and BOC(6,1) in the pilot component are added in antiphase (−). The combination of both sub-carriers is the so-called CBOC modulation. Then, the I/NAV navigation data message is first modulated by the ranging code and then by the two sub-carriers, each with a different weighting coefficient $\alpha =\sqrt{\frac{10}{11}}$ and $\beta =\sqrt{\frac{1}{11}}$. In parallel, the pilot component ranging code is also modulated by these sub-carriers.
- *Galileo E6 Signal*: The signal transmitted in the Galileo E6 band has two components: the data component which transmits the navigation message (with a symbol rate of 1000 symbols/s), called C/NAV message [24], and the pilot component. For the data component, the PRN sequence of 5115 chips lasts 1 ms, and for the pilot (combination with a secondary code of length 100) 100 ms. This signal is QPSK(5) modulated, that is, in-phase and quadrature BSPK(5) signals.
- *Galileo E5 Signal*: Within the Galileo E5 band, the Galileo System generates and broadcasts the E5 signal. This signal is separated with four signal components and it is allocated in two different frequency sub-bands, denoted as E5a (1176.45 MHz) and E5b (1207.14 MHz). Within each sub-band, one data component (in-phase) and one pilot component (quadrature) are transmitted [24]. The F/NAV (E5a-I) and I/NAV (E5b-I) navigation messages [24] are transmitted at 50 symbols/s and 250 symbols/s. The E5a-I and E5b-I PRN (combination with a secondary code of length 20 and 4, respectively) last 20 and 4 ms. The quadrature components PRN (combination with a secondary code of length 100) lasts 100 ms. The Galileo E5 signal is constructed as an AltBOC(15,10,10), or simply denoted as AltBOC(15,10), modulated signal. Refer to Section 6.1 for details on the AltBOC modulation.

**Figure 2:**
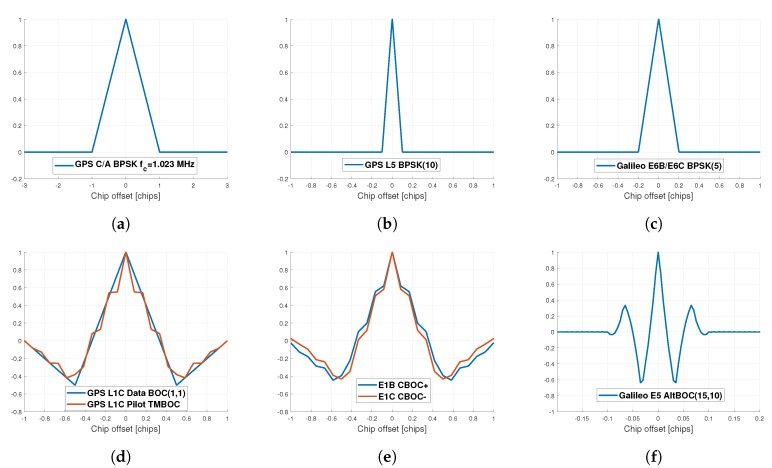
ACF of the GPS L1 C/A (**a**), GPS L5 (**b**), Galileo E6 (**c**), GPS L1C (**d**), Galileo E1 OS (**e**) and Galileo E5 (**f**).

The ACFs for the different modulations, from the GPS L1 C/A BPSK(1) to the Galileo E5 AltBOC(15,10), are shown in Figure 2.

## 6. GNSS Meta-Signals

The concept of *GNSS meta-signals* was introduced for the first time in [31]. A GNSS meta-signal is the combination of two different GNSS signals transmitted at two different carrier frequencies which can be expressed as a single Alternate Binary Offset Carrier (AltBOC) modulated signal [32]. Based on that initial work, Paonni et al. [33] further discussed the fundamental concept of GNSS meta-signals, proving through analytical and practical implementations that, unlike other conventional methods, where GNSS receivers process multiple signals, the GNSS meta-signal method could improve the single-point ranging accuracy over that of the better of the two generating signals. Two different GNSS meta-signals have been proposed [31,33] considering Galileo signals

- Galileo E5a + E6-BC: AltBOC(50,10,5) [31,33],
- Galileo E5b + E6-BC: AltBOC(35,10,5) [31,33],

In both works [31,33], the GNSS meta-signal built from the combination of the Galileo E5b and Galileo E6-BC signals is shown to be especially interesting. This is because this specific GNSS meta-signal is centered at 1242.925 MHz which is exactly half frequency of the carrier frequency of a potential GNSS signal in the S-band [31].

### 6.1. Generalized AltBOC

The AltBOC modulation was original introduced as a method to combine Galileo signals within the E5a and E5b bands. This solution was rapidly accepted by the Galileo Signal Task Force (GSTF) since the AltBOC modulation: (i) provides a Constant Envelope Modulation (CEM), which avoids non-linear distortions at the output of the High Power Amplifier (HPA); and (ii) it provides a high level of isolation between two frequency bands [32]. The easiest form of AltBOC modulation is the one where two independent PRN codes are multiplexed. Let us define the BOC subcarrier with cosine and sine phasing as $SC_{cos}\left(t\right)=\mathrm{sign}\left(\cos\left(2\pi F_{sub}t\right)\right)$ and $SC_{sin}\left(t\right)=\mathrm{sign}\left(\sin\left(2\pi F_{sub}t\right)\right)$, respectively, where $F_{sub}$ represents the subcarrier frequency. Then, we can built the Single Side Band (SSB) subcarrier $SC_{SSB}$ and its conjugate $SC_{SSB}^{*}$ as,

(12) $$\begin{array}{c} SC_{4,SSB}\left(t\right)=\frac{1}{\sqrt{2}}\left(SC_{cos}\left(t\right)+j\cdot SC_{sin}\left(t\right)\right)\;;\;SC_{4,SSB}^{*}\left(t\right)=\frac{1}{\sqrt{2}}\left(SC_{cos}\left(t\right)-j\cdot SC_{sin}\left(t\right)\right). \end{array}$$

Note that Equation (12) can take four values and it can be also derived as,

(13) $$\left\{\begin{array}{ccc} SC_{SSB}\left(t\right) & = & e^{j\left(\frac{\pi }{4}+i\cdot \frac{\pi }{2}\right)};\\ t\;\mathrm{mod}\;T_{sub} & \in & \left[\frac{i\cdot T_{s}}{4},\frac{\left(i+1\right)\cdot T_{sub}}{4}\right], \end{array}\right.$$

with $i\in \left[0,1,2,3\right]$ and $T_{sub}=1/F_{sub}$. Finally, the two-code AltBOC can be defined as,

(14) $$\begin{array}{cc} c(t) & =c_{A}\left(t\right)SC_{4,SSB}^{*}\left(t\right)+c_{B}\left(t\right)SC_{4,SSB}\left(t\right)=\left[c_{A}\left(t\right)+c_{B}\left(t\right)\right]SC_{cos}\left(t\right)+j\cdot \left[c_{B}\left(t\right)-c_{A}\left(t\right)\right]SC_{sin}\left(t\right) \end{array}$$

where $c_{A}\left(t\right)$ and $c_{B}\left(t\right)$ represent binary PRN codes of the low (*A*) and high (*B*) band signals, respectively. Note from the previous equation that codes $c_{A}\left(t\right)$ and $c_{B}\left(t\right)$ are not required to have the same chip rate. Considering two independent codes (in-phase/quadrature) per each frequency band, a four-code AltBOC modulation can be expressed as,

(15) $$\begin{array}{cc} c(t) & =\left[c_{A,I}\left(t\right)+j\cdot c_{A,Q}\left(t\right)\right]SC_{4,SSB}^{*}\left(t\right)+\left[c_{B,I}\left(t\right)+j\cdot c_{B,Q}\left(t\right)\right]SC_{4,SSB}\left(t\right)\;, \end{array}$$

where $c_{A,I}\left(t\right)$ and $c_{A,Q}\left(t\right)$ represent binary in-phase/quadrature codes of the *A* band and $c_{B,I}\left(t\right)$ and $c_{B,Q}\left(t\right)$ represent binary in-phase/quadrature codes of the *B* band. The resulting constellation from Equation (15) was shown by Lestarquit et al. [32] to not a have constant envelope and consequently such modulation cannot be used with HPA working at the saturation point. In order to obtain a CEM, an intermodulation product [34] must be added within the composite signal, yielding Equation (15) to [35],

(16) $$\begin{array}{cc} c(t) & =\left[c_{A,I}\left(t\right)+j\cdot c_{A,Q}\left(t\right)\right]SC_{8,SSB}^{*}\left(t\right)+\left[c_{B,I}\left(t\right)+j\cdot c_{B,Q}\left(t\right)\right]SC_{8,SSB}\left(t\right)\\ \; & +\left[\bar{c_{A,I}\left(t\right)}+j\cdot \bar{c_{A,Q}\left(t\right)}\right]SC_{P,8,SSB}^{*}\left(t\right)+\left[\bar{c_{B,I}\left(t\right)}+j\cdot \bar{c_{B,Q}\left(t\right)}\right]SC_{P,8,SSB}\left(t\right) \end{array}$$

where $\bar{c_{A,I}\left(t\right)}=c_{A,Q}\left(t\right)c_{B,I}\left(t\right)c_{B,Q}\left(t\right)$, $\bar{c_{A,Q}\left(t\right)}=c_{A,I}\left(t\right)c_{B,I}\left(t\right)c_{B,Q}\left(t\right)$, $\bar{c_{B,I}\left(t\right)}=c_{B,Q}\left(t\right)c_{A,I}\left(t\right)c_{A,Q}\left(t\right)$ and $\bar{c_{B,Q}\left(t\right)}=c_{B,I}\left(t\right)c_{A,I}\left(t\right)c_{A,Q}\left(t\right)$, and $SC_{8,SSB}\left(t\right)$ is the SSB “single” subcarrier of the four-code AltBOC. Moreover, $SC_{P,8,SSB}\left(t\right)$ is defined as the “product” subcarrier. Both subcarriers can take eight different values [32],

(17) $$\left\{\begin{array}{ccc} SC_{8,SSB}\left(t\right) & = & e^{j\left(\frac{\pi }{8}+i\cdot \frac{\pi }{4}\right)};\\ t\;\mathrm{mod}\;T_{sub} & \in & \left[\frac{i\cdot T_{sub}}{8},\frac{\left(i+1\right)\cdot T_{s}}{8}\right], \end{array}\right.\;;\;\left\{\begin{array}{ccc} SC_{P,8,SSB}\left(t\right) & = & e^{j\left(\frac{5\pi }{8}-i\cdot \frac{3\pi }{4}\right)};\\ t\;\mathrm{mod}\;T_{sub} & \in & \left[\frac{i\cdot T_{sub}}{8},\frac{\left(i+1\right)\cdot T_{s}}{8}\right], \end{array}\right.$$

with $i\in \left[0,1,2,3,4,5,6,7\right]$. Note from Lestarquit et al. [32] that most of the energy of $SC_{P,8,SSB}\left(t\right)$ and $SC_{P,8,SSB}^{*}\left(t\right)$ is located in the fundamental harmonics, $F_{sub}$ and $-F_{sub}$, respectively. On the other hand, the energy of $SC_{P,8,SSB}\left(t\right)$ and $SC_{P,8,SSB}^{*}\left(t\right)$ is located in the harmonics $-3F_{sub}$, $5F_{sub}$ and $3F_{sub}$, $-5F_{sub}$, respectively.

### 6.2. AltBOC Spectral and Correlation Properties

If one wants to compute the AltBOC PSD, it is interesting to use the signal formulation introduced in [30]. According to Rodríguez [30], any chip within the PRN code can be seen as a number of equal-length deterministic segments with different amplitude levels (also known as Multilevel Coded Spreading (MCS) symbols). Then, the expression of the chip waveform is $p_{chip}\left(t\right)=\sum_{n=0}^{N_{x}-1}a_{n}p_{sub_{chip}}\left(t-n\frac{T_{c}}{N_{x}}\right)$, where $N_{x}$ is the number of equal-length segments, i.e., sub-chips, and $p_{sub_{chip}}$ represents the sub-chip shape. Note that in the GNSS framework $p_{sub_{chip}}$ is usually assumed to be rectangular. Moreover, $\frac{T_{c}}{N_{x}}$ can also be defined as the subcarrier period $T_{sub}$. The transmitted signal is $c\left(t\right)=\sum_{n=-\infty }^{+\infty }c_{n}p_{chip}\left(t-nT_{c}\right)$, with $c_{n}$ the amplitude of the PRN code sequence. Considering that the PRN code shows ideal statistical properties, the PSD of c(t) simplifies to $G_{c}\left(f\right)=\left|C\left(f\right)\right|/T_{c}$ [30],

(18) $$G_{c}\left(f\right)=\frac{1}{T_{c}}\frac{\sin^{2}\left(\frac{\pi fT_{c}}{N_{x}}\right)}{{\left(\pi f\right)}^{2}}{\left|\left|\sum_{n=1}^{N_{x}}a_{n}e^{-2\pi j\frac{nfT_{c}}{N_{x}}}\right|\right|}^{2}.$$

where the first term defines the PSD of a Binary Phase Shift Keying (BPSK) modulation with chip rate $\frac{T_{c}}{N_{x}}$ and the second term determines the MCS modulation family, $G_{c}\left(f\right)=G_{BPSK}\left(f\right)G_{mod}\left(f\right)$. Assuming that the AltBOC transmits codes with the same chip rate (symmetrical AltBOC), one can use the approach proposed in [36] to compute the symmetrical AltBOC PSD. Since GNSS meta-signals may be generated by non-symmetrical AltBOC waveforms, i.e., the chip rate of the upper and lower codes are not the same, we follow the approach Paonni et al. [33] to compute the GNSS meta-signals PSD. We use the notation AltBOC(p,q,w), where $F_{sub}=pf_{0}$ is the subcarrier frequency with $f_{0}=1.023$ MHz and $f_{c,A}=qf_{0}$ and $f_{c,B}=wf_{0}$ are the lower and upper codes’ chip rate, respectively. The non-symmetrical AltBOC PSD expression is therefore [33],

(19) $$G_{AltBOC(p,q,w)}\left(f\right)=4\left[\begin{array}{c} G_{BPSK,N_{A}}\left(f\right)G_{mod,N_{A}}\left(f\right)+G_{BPSK,N_{B}}\left(f\right)G_{mod,N_{B}}\left(f\right)\\ +G_{BPSK,N_{\bar{A}}}\left(f\right)G_{mod,N_{\bar{A}}}\left(f\right)+G_{BPSK,N_{\bar{B}}}\left(f\right)G_{mod,N_{\bar{B}}}\left(f\right) \end{array}\right],$$

where

(20) $$\begin{array}{cc} \; & G_{BPSK,N_{A}}\left(f\right)=f_{c,A}\frac{sin^{2}\left(\frac{\pi f}{N_{A}f_{c,A}}\right)}{{\left(\pi f\right)}^{2}},\;G_{BPSK,N_{B}}\left(f\right)=f_{c,B}\frac{sin^{2}\left(\frac{\pi f}{N_{B}f_{c,B}}\right)}{{\left(\pi f\right)}^{2}}, \end{array}$$

(21) $$\begin{array}{cc} \; & G_{BPSK,N_{\bar{A}}}\left(f\right)=f_{c,\bar{A}}\frac{sin^{2}\left(\frac{\pi f}{N_{\bar{A}}f_{c,\bar{A}}}\right)}{{\left(\pi f\right)}^{2}}, \end{array}$$

with $G_{BPSK,N_{\bar{A}}}\left(f\right)=G_{BPSK,N_{\bar{B}}}\left(f\right)$, $N_{A}=4\left(2p/q\right)$, $N_{B}=4\left(2p/w\right)$, $N_{\bar{A}}=N_{\bar{B}}=4\left(2p/\mathrm{LCM}(q,w)\right)$ and $f_{c,\bar{A}}=\mathrm{LCM}\left(f_{c,A},f_{c,B}\right)$. Note that LCM stands for Least Common Multiple. In addition,

(22) $$\begin{array}{cc} \; & G_{mod,N_{A}}\left(f\right)={\left|\left|\sum_{n=1}^{N_{sub}}a_{n_{A}}e^{-2\pi j\frac{nf}{N_{A}f_{c,A}}}\right|\right|}^{2}\;;\;G_{mod,N_{B}}\left(f\right)={\left|\left|\sum_{n=1}^{N_{sub}}a_{n_{B}}e^{-2\pi j\frac{nf}{N_{B}f_{c,B}}}\right|\right|}^{2}, \end{array}$$

(23) $$\begin{array}{cc} \; & G_{mod,N_{\bar{A}}}\left(f\right)={\left|\left|\sum_{n=1}^{N_{sub}}a_{n_{\bar{A}}}e^{-2\pi j\frac{nf}{N_{\bar{A}}f_{c,\bar{A}}}}\right|\right|}^{2}\;;\;G_{mod,N_{\bar{B}}}\left(f\right)={\left|\left|\sum_{n=1}^{N_{sub}}a_{n_{\bar{B}}}e^{-2\pi j\frac{nf}{N_{\bar{A}}f_{c,\bar{A}}}}\right|\right|}^{2}, \end{array}$$

where $a_{n_{A}}$, $a_{n_{B}}$, $a_{n_{\bar{A}}}$, and $a_{n_{\bar{B}}}$ are the complex values derived from $SC_{8,SSB}^{*}\left(t\right)$, $SC_{8,SSB}\left(t\right)$, $SC_{P,8,SSB}^{*}\left(t\right)$, and $SC_{P,8,SSB}\left(t\right)$, respectively (refer to Equation (17)).

**Figure 3:**
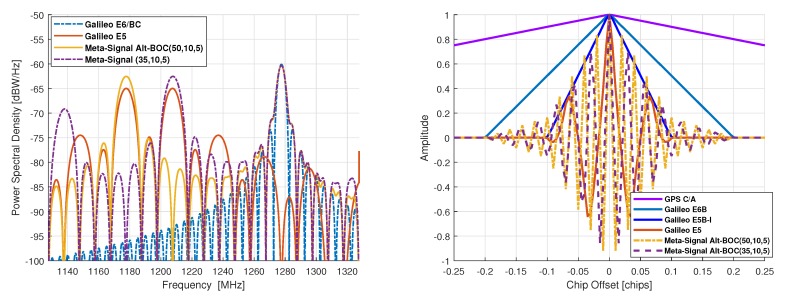
(**Left**) Galileo E5/E6 and GNSS meta-signal PSD, and (**Right)** GPS L1 C/A, Galileo E6B, E5b-I, E5 and GNSS meta-signals ACFs, *with intermodulation products*. Notice the width of the E6B ACF in $\left[-\frac{T_{c}}{5},\frac{T_{c}}{5}\right]$ because it uses a BPSK(5), and the E5b-I ACF width in $\left[-\frac{T_{c}}{10},\frac{T_{c}}{10}\right]$ because it uses a BPSK(10).

Finally, define the autocorrelation function in terms of the PSD, we resort to the Wiener–Khintchine theorem [37] (Chapter 10), which states that the ACF and its PSD function are a Fourier transform pair defined as,

(24) $$ACF\left(t\right)=\int_{-\frac{Br}{2}}^{\frac{Br}{2}}G_{c}\left(f\right)e^{-j2\pi ft}df\;,$$

with Br the receiver bandwidth. The PSD of Galileo E5/E6 signals along with the GNSS meta-signal PSD defined by the AltBOC(50,10,5) and AltBOC(35,10,5) are illustrated in Figure 3 (left). Note from these results that the PSD of GNSS meta-signals slightly differ from the PSD of Galileo E5/E6. This is because the AltBOC modulation adds intermodulation products (refer to Equation (16)) to keep the constant envelope shape. Note also that generating those extra intermodulation products at the receiver replica is unnecessary since they were not generated at the transmission engine (Galileo E5 and E6 are transmitted with two independent HPA amplifiers). The corresponding ACFs are illustrated in Figure 3 (right), which shows that ACFs of GNSS meta-signals are narrower than those obtained for E5/E6. For completeness, we computed the ACF of the meta-signals *without intermodulation products*, but the difference with the ACF shown in Figure 3 is marginal, with no impact on the delay estimation accuracy.

A summary of the different GNSS signals’ main characteristics is given in Table 1.

## 7. Results: Time-Delay Estimation Accuracy Limit for Some Representative GNSS Signals

To assess the time-delay performance limit with the different GPS, Galileo and GNSS meta-signals introduced in the previous sections, we computed the closed-form CRB in Equation (6) and the corresponding MLE in Equation (3) for these GNSS representative signals:

- the 1 ms of the GPS L1 C/A with PRN codes of length 1023 chips, BPSK(1);
- the 10 ms for the GPS L1C (data) with a PRN code of length 10,230 chips and a BOC(1,1) subcarrier;
- the 10 ms of the GPS L1C (pilot) with a PRN code of length 10,230 chips and a TMBOC subcarrier;
- the 4 ms of the Galileo E1B (data) with a PRN code of length 4096 chips and a CBOC(+) subcarrier;
- the 4 ms of the Galileo E1C (pilot) with a PRN code of length 4096 chips and a CBOC(−) subcarrier;
- the 1 ms of the GPS L5-I with PRN codes of length 10,230 chips, BPSK(10);
- the 1 ms of the Galileo E6B signal with PRN code of length 5115 chips, BPSK(5);
- the 1 ms of the Galileo E5b-I with PRN code of length 10,230 chips, BPSK(10);
- the 1 ms of the Galileo E5 AltBOC(15,10) with PRN codes of length 10230 chips;
- the 1 ms of the meta E5b + E6-BC AltBOC(35,10,5) signal, with PRNs of length 10,230 and 5115 chips; and
- the 1 ms of the meta E5a + E6-BC AltBOC(50,10,5) signal, with PRNs of length 10,230 and 5115 chips.

The CRB in Equation (6) and the corresponding MLE in Equation (3) were computed considering $\alpha =\left(1+j\right)\cdot \sqrt{SNR_{in}/2}$. The MLE was obtained from 1000 Monte Carlo runs. Section 7.1 shows the comparison of the legacy L1 C/A with the new GPS L1C and the Galileo E1 OS (E1B and E1C), the comparison with the other GPS civil signals at L2 and L5 is shown in Section 7.2, and the complete discussion with the other Galileo signals and meta-signals in Section 7.3. Notice that the SNR${}_{\mathrm{out}}$ in the following results refers to the SNR at the output of the matched filter. The maximum SNR at the output of the ML matched filter is

(25) $$\begin{array}{cc} \; & {\mathrm{SNR}}_{\mathrm{out}}=\frac{F_{s}{\left|\alpha \right|}^{2}c^{H}c}{\sigma_{n}^{2}}=\frac{C}{N_{0}}T_{\mathrm{PRN}}L_{c}, \end{array}$$

where $C/N_{0}$ (dB-Hz) is the carrier-to-noise density ratio, $T_{\mathrm{PRN}}$ is the single code duration (equal to 1 ms in the case of GPS L1 C/A), and $L_{c}$ is the number of codes; therefore, $T_{I}=T_{\mathrm{PRN}}\times L_{c}$ is the coherent integration time. Then, we could verify that SNR${}_{\mathrm{out}}=25$ dB and $T_{I}=10$ ms imply a $C/N_{0}=45$ dB-Hz, which is a nominal GNSS value. In addition, the MLE threshold, i.e., the point where the MLE reaches the optimal operation regime, was always found around SNR${}_{\mathrm{out}}=15$ dB, which for $T_{I}=1$ ms corresponds to a $C/N_{0}=45$ dB-Hz, for $T_{I}=10$ ms to a $C/N_{0}=35$ dB-Hz, and for $T_{I}=20$ ms to a $C/N_{0}=32$ dB-Hz. The latter fixes the limit for the use of the GNSS signals in standard coherent integration architectures. To go below this $C/N_{0}$ value, we have to resort to the so-called high-sensitivity GNSS (HS-GNSS) techniques, with combinations of coherent and non-coherent integrations due to the navigation data bits.

### 7.1. GPS L1 C/A vs. GPS L1C and Galileo E1 Open Service

**Figure 4:**
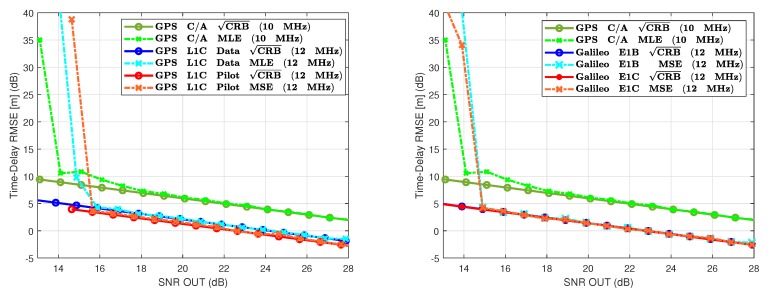
(**Left**) CRB/MLE for GPS L1 C/A BPSK(1), GPS L1C data component BOC(1,1), and GPS L1C pilot component TMBOC. (**Right**) CRB/MLE for GPS L1 C/A BPSK(1), Galileo E1B CBOC(+), and Galileo E1B CBOC(−).

First, we assessed the CRB for the GPS and Galileo civil signals broadcasted in the L1 band. Figure 4 summarizes the results for both time-delay CRB and MLE, the left panel for the GPS L1 C/A vs. GPS L1C comparison and the right panel for the GPS L1 C/A vs. Galileo E1B/E1C one. Notice that we used a sampling rate $F_{s}=10$ MHz for the GPS L1 C/A, $F_{s}=12$ MHz for both GPS L1C pilot and data components, and $F_{s}=12$ MHz for both E1B and E1C components.

A first look at the CRB results in Figure 4 clearly show the improvement on the time-delay estimation performance between the GPS L1C or Galileo E1 OS with respect to the GPS C/A. This is mainly due to the subcarrier modulation, that is, GPS L1C and Galileo E1 OS (data and pilot components) are modulated by BOC-type subcarriers, which results in narrower ACFs. In addition, these new L1 GNSS signals have longer PRN codes; consequently, for the same chip rate, the coherent integration time is longer, which translates to a higher SNR${}_{\mathrm{out}}$ operation point. We also can see a slight improvement of the GPS L1C pilot component with respect to the GPS L1C data component. Again, this is mainly due to the ACF shape, which is narrower for the TMBOC modulation than for the BOC(1,1). Moreover, at the signal generation point, 75% of the power is dedicated to generate the pilot component of the GPS L1C signal, while only 25% is used to generate the GPS L1C data component. In the Galileo E1 OS case, we obtain the same results for both E1B and E1C signals.

Comparing these results at a particular operation point, that is, SNR${}_{\mathrm{out}}=25$ dB, we obtained the following time-delay standard deviation: (i) 2.24 m for the legacy GPS L1 C/A; (ii) 92 cm for the GPS L1C data component; (iii) 75 cm for the GPS L1C pilot component; (iv) 78 cm for the Galileo E1B data component; and (v) 77 cm for the Galileo E1C pilot component. Then, using these BOC-type subcarrier modulations, we gain around a factor 2.5 on the standard deviation, but still we are far from calling it a precise positioning solution.

### 7.2. GPS L1 C/A vs. GPS L5

**Figure 5:**
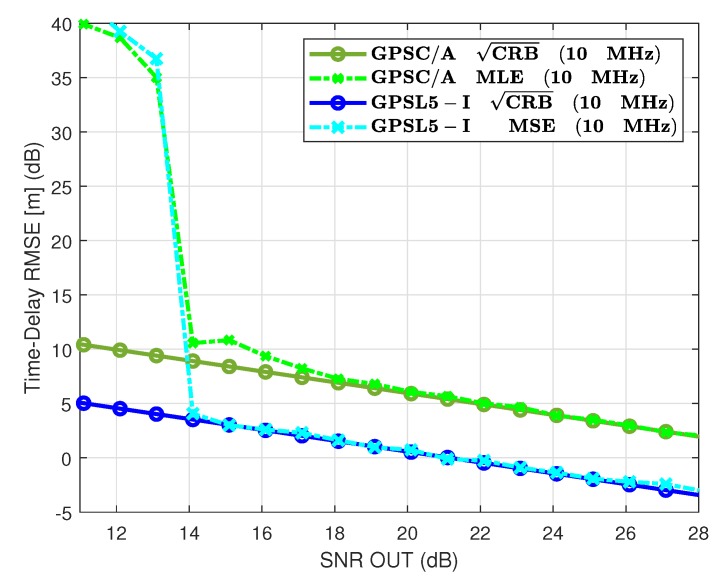
CRB/MLE for GPS L1 C/A BPSK(1) and GPS L5-I BPSK(10).

The other GPS civil signals do not exploit BOC-type modulations but in contrast use: (i) a BPSK(10) for the GPS L5; (ii) a BPSK(0.5) for the GPS L2C if we take one of the two codes; or (iii) a BPSK(1) for the GPS L2C considering both CM and CL codes. Then, the time-delay performance obtained with the L2C is either the same or slightly lower than the one obtained with the legacy L1 C/A signal. The CRB and MLE results in Figure 5 show that using such fast BPSK obviously also improves the time-delay estimation capabilities. Considering again the SNR${}_{\mathrm{out}}=25$ dB operation point, we have now for the GPS L5 a standard deviation of 64 cm, being even better than the GPS L1C and Galileo E1 OS components. However, again, a 3σ error of 1.9 m cannot be seen as a precise positioning solution.

### 7.3. GPS L1 C/A vs. Galileo E6B, E5b, E5 and GNSS Meta-Signals

**Figure 6:**
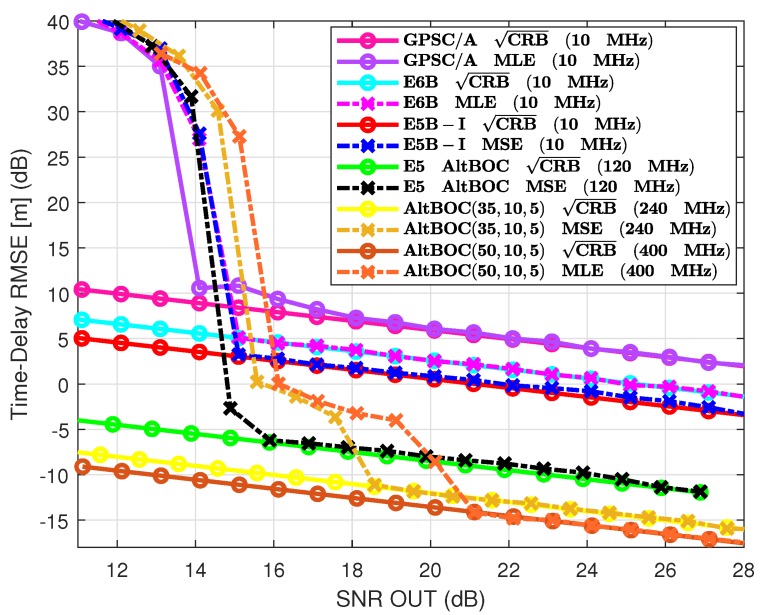
CRB/MLE for GPS L1 C/A BPSK(1), E6B BPSK(5), E5b-I BPSK(10), E5 AltBOC(15,10), meta-signal E5b/E6 AltBOC(35,10,5), and meta-signal E5a/E6 AltBOC(50,10,5).

To complete the analysis, we summarize in Figure 6 the results obtained with the other Galileo signals, i.e., E6B, E5b-I, and E5, together with the ones obtained for the Galileo E5b/E6B and E5a/E6 meta-signals, that is, the AltBOC(35,10,5) and AltBOC(50,10,5), respectively. Recall that from the previous subsections the benchmark results (at SNR${}_{\mathrm{out}}=25$ dB) are: (i) standard deviation of 2.24 m for the legacy GPS L1 C/A and $F_{s}=10$ MHz; (ii) standard deviation of 75 cm for the GPS L1C pilot and $F_{s}=12$ MHz; and (iii) standard deviation of 64 cm for the GPS L5 and $F_{s}=10$ MHz. Using the E6B or E5b-I signals gives at the same operation point a standard deviation equal to 1 m and 64 cm, respectively, which is obvious considering the underlying BPSK(5) and BPSK(10) modulations. Notice that because the time-delay estimation performance is driven by the signal modulation, GPS L5 and Galileo E5b or Galileo E5a are equivalent in terms of estimation performance.

What is remarkable is the performance improvement provided by the Galileo E5 AltBOC(15,10) if we exploit the complete bandwidth, which at this particular operation point provides an estimate with a standard deviation equal to 8 cm. Considering now the results obtained with the Galileo E5b-E6B AltBOC(35,10,5) and E5a-E6 AltBOC(50,10,5) meta-signals, and exploiting the complete bandwidth, we obtained a further improvement, with a standard deviation for the E5b-E6B of 3.5 cm, and for the E5a-E6 equal to 2.5 cm. These AltBOC-modulated signals can be considered as an option to precise positioning, with 3σ delay errors below 25 cm, i.e., below 12 cm for the meta-signals.

It is important to notice the behavior of the MLE between the previous 15 dB threshold and the convergence to the CRB. This is because of the special shape of the ACF, as shown in Figure 3. With respect to the E5, these meta-signals have positive secondary ACF peaks which are very close to the main peak. These secondary peaks induce a second operation region of the MLE between the CRB and the threshold. Indeed, in this intermediate operation region, the MLE jumps between secondary peaks because of the noise, which introduces estimation errors. Note that this is the effect known as false locks in high-order BOC signals such as the BOC(15,2.5) used in the Galileo E1 PRS. Then, the use of meta-signals can improve the delay estimation with respect to the Galileo E5a, E5b, E6, and the complete E5 signals if the receiver is able to cope with such false locks or the SNR at the output of the matched filter is high enough. In addition, to reach such centimeter-level accuracy the receiver needs to exploit a huge bandwidth; thus, depending on the application and design constraints, a wise choice may be to use the E5 AltBOC(15,10). To summarize, the E5 signal can reduce the delay standard deviation by a factor of 28, 12, and 8 with respect to the GPS L1 C/A, Galileo E6B, and Galileo E5b-I/GPS L5 signals, respectively. An additional factor of 2.5 and 3.5 can be obtained by means of meta-signals. These results confirm that AltBOC-type signals can provide decimetric accuracy, thus being a promising solution (and the only one) for code-based precise positioning.

### 7.4. Theoretical Performance Limits of AltBOC-Type GNSS Signals

A remaining point is to assess the theoretical performance limits of the promising AltBOC-type signals, that is, which is the ultimate achievable time-delay estimation performance for higher SNR at the output of the matched filter and which are the conditions which allow to achieve such results. From the previous results, we know that the MLE for these types of signals is efficient if the SNR${}_{\mathrm{out}}>16$ dB for the E5, SNR${}_{\mathrm{out}}>19$ dB for the AltBOC(35,10,5), and SNR${}_{\mathrm{out}}>21$ dB for the AltBOC(50,10,5); thus, in the following, we do not plot the MLE. Notice that, to be able to increase the SNR at the output of the MLE, we must increase the coherent integration time (refer to Equation (25)), but the E5 is limited to the 4 ms data bits, and the meta-signals are limited to the 1 ms bits of the E6. Then, let us assume that we can obtain the navigation message by other means, perform a data wipe-off, and therefore use longer coherent integration time periods. In this case, for a $C/N_{0}=45$ dB-Hz, we can obtain a SNR${}_{\mathrm{out}}=30,35,40,45$, and 50 dB by roughly integrating 30 ms, 100 ms, 300 ms, 1 s, and 3 s, respectively. For Galileo signals, the nominal $C/N_{0}$ is considered around 50 dB-Hz, which translates to an integration time roughly equal to 10 ms, 35 ms, 100 ms, 350 ms, and 1 s, respectively. Notice that these values are the typical ones considered in the context of HS-GNSS receivers [38] to be able to operate at very low $C/N_{0}$ such as indoors or in space exploration. The standard deviation results obtained with such long integration times are summarized in Table 2. This is an important result, which shows that we can go below the time-delay cm accuracy if we adopt long coherent integration times.

### 7.5. Performance Limits of Phase Estimation with AltBOC-Type GNSS Signals

As already stated in the Introduction, the standard way to obtain GNSS precise positioning solutions is to exploit the signal phase information via RTK or PPP techniques [9]. The main problem with these techniques is that they have to deal with phase ambiguities, which imply long convergence times for PPP and complicated ambiguity resolution techniques for RTK, in addition to being limited to a low number of satellites to achieve high fixing success rates [9] (Chapter 23). From the CRB results in Section 3, while the time-delay estimation is related to the baseband signal resolution, as shown in the previous subsections for the different GPS and Galileo signals/meta-signals, phase estimation is mainly driven by the SNR at the output of the matched filter.

Indeed, the phase estimation CRB is ${\mathrm{CRB}}_{\varphi |\underline{\epsilon }}≃\frac{1}{2{\mathrm{SNR}}_{\mathrm{out}}}$ (equality for real signals). Then, using fast codes such the ones in GPS L5 or Galileo E6, E5a, E5b, or AltBOC-type signals as in Galileo E5 and GNSS meta-signals, do not improve the phase estimation with respect to the legacy GPS L1 C/A signal. Then, the huge advantage of using these new signals in terms of time-delay estimation precision does not translate to the corresponding signal phase. This is an additional noteworthy result to further support the fact that AltBOC-type signals can be a promising solution for code-based precise positioning, because the advantage brought by joint delay/phase positioning using these signals may not be worth the inherent complexity and limitations of ambiguity resolution techniques. Let us recall that the MLE of time-delay and phase are given by

(26) $$\begin{array}{cc} \; & \hat{\tau }=\arg\max_{\tau }\left\{{\left|{\left(c{\left(\tau \right)}^{H}c\left(\tau \right)\right)}^{-1}c{\left(\tau \right)}^{H}x\right|}^{2}\right\}, \end{array}$$

(27) $$\begin{array}{cc} \; & \hat{\varphi }\left(\hat{\tau }\right)=\arg\left\{{\left(c{\left(\hat{\tau }\right)}^{H}c\left(\hat{\tau }\right)\right)}^{-1}c{\left(\hat{\tau }\right)}^{H}x\right\}, \end{array}$$

then the phase is estimated as the argument of the cross-ambiguity function evaluated at the time-delay MLE. Therefore, we are only concerned by the receiver operation points where we obtain a correct delay estimate, that is, $15<{\mathrm{SNR}}_{\mathrm{out}}<28$ dB (the upper value given by $T_{I}=20$ ms at a nominal $C/N_{0}=45$ dB-Hz, which is the $T_{\mathrm{bit}}$ coherent integration time limit in GPS L1 C/A signals without considering HS-GNSS architectures). In Table 3, we give some phase estimation precision results and the corresponding time-delay ones. Notice that $\hat{\varphi }\left(\hat{\tau }\right)$ in meters was obtained as $\frac{\lambda }{2\pi }\sqrt{{\mathrm{CRB}}_{\varphi |\underline{\epsilon }}}$. The values for different wavelengths are almost the same (i.e., $\lambda_{L1}=19.03$ cm, $\lambda_{L5}=25.48$ cm, $\lambda_{E5}=25.15$ cm, $\lambda_{E5b-E6}=24.12$ cm and $\lambda_{E5a-E6}=24.42$ cm), thus we use $\lambda_{E5}$ in Table 3.

From the GPS L1 C/A results shown in Table 3, we see that we obtain the typical values found in the GNSS literature, that is, code observable standard deviation $\sigma_{\rho_{L1}}≃\left[2–5\right]$ m and phase observable standard deviation $\sigma_{\varphi_{L1}}≃\left[1–3\right]$ mm. Using AltBOC-type signals, we reduce $\sigma_{\rho_{L1}}$ by a factor of 27; thus, the difference between code, i.e., delay, and phase observables standard deviation is reduced from a factor around 2500 (L1 C/A) to a factor around 100 (AltBOC signals). Therefore, using PPP or RTK techniques in the context of AltBOC-type signals is much less interesting than for standard signals, which supports the previous discussion on code-based AltBOC PPP or RTK.

## 8. Conclusions and Outlook

In this contribution, we propose to exploit a recently derived compact time-delay estimation CRB which depends only on the signal samples. This is especially useful to correctly evaluate different signals and obtain meaningful results. The CRB and the corresponding MLE were computed for a set of representative GNSS signals: the legacy GPS L1 C/A; the other GPS civil signals L1C and L5; the family of Galileo signals E1 OS, E6, E5a, E5b, and E5; and the new GNSS meta-signal paradigm with the combination of the E6 with both E5a and E5b. The goal of this contribution was to determine: (i) the ultimate achievable performance of GNSS code-based positioning systems; and (ii) whether we could obtain a GNSS code-only precise positioning solution and under which conditions.

Regarding the first question, at a representative SNR${}_{\mathrm{out}}=25$ dB operation point at the output of the MLE, it was found that the time-delay standard deviation using BPSK and BOC-type signals ranges between 2.24 m for GPS L1 C/A and 64 cm for the GPS L5 or the individual E5 components, i.e., E5a or E5b. Using the complete E5 signal, we can reduce the delay standard deviation by a factor 28, 12, and 8 with respect to the GPS L1 C/A, Galileo E6B, and Galileo E5b-I/GPS L5 signals, respectively, that is, a standard deviation of 8 cm. This performance can be further improved by an additional factor of 2 and 3 using the E5b-E6 and E5a-E6 meta-signals, for which we obtained 3.5 and 2.5 cm, respectively. The latter comes at the expense of possible false locks due to high secondary correlation peaks and a huge bandwidth. With respect to the second question, only the AltBOC-type signals can provide decimetric precision, thus only the E5 and GNSS meta-signals can be considered as an option for code-based precise positioning. For instance, using the complete E5 signal, the 3σ time-delay error is below 25 cm. Obviously, to reach such precisions, we still need to be able to correct for external errors such as ionospheric/tropospheric delays, as well as orbital or satellite clock errors as in PPP solutions, but this could also be exploited to avoid carrier phase ambiguity fixing in code-based RTK solutions. The latter may be a promising line to be explored, because such ambiguity fixing is essentially the bottleneck of RTK techniques, and is still an open issue when using a large number of satellites in multi-constellation/multi-frequency architectures. In addition, exploiting long coherent integration times, we may be able to reach code-based sub-cm accuracy. Finally, we also derived a new sample-based joint delay/phase estimation CRB, which was used to characterize the possible performance improvement of phase-based techniques in the AltBOC-type signals context. It was found that the phase CRB do not depend on the signal, and therefore the gain provided by the phase exploitation must not be worth the ambiguity resolution complexity in AltBOC-type architectures. The results presented in this contribution open the door to new precise positioning receiver design.

## Figures and Tables

**Table 1:** GPS, Galileo, and meta-signals main characteristics. $T_{c}$ is defined as the GPS L1 C/A chip period, i.e., 1.023 × 10${}^{-6}$ s, and ACF main peak refers to the first zero-crossing of the ACF.

Signal	Modulation	$T_{\mathrm{PRN}}$ (ms)	$T_{\mathrm{bit}}$ (ms)	ACF Main Peak
GPS L1 C/A	BPSK(1)	1	20	$\pm T_{c}$
GPS L1C Data	BOC(1,1)	10	10	$\pm 0.33T_{c}$
GPS L1C Pilot	TMBOC	10	-	$\pm 0.35T_{c}$
GPS L5-I	BPSK(10)	1	10	$\pm 0.1T_{c}$
GPS L5-Q	BPSK(10)	1	-	$\pm 0.1T_{c}$
Galileo E1B	CBOC +	4	4	$\pm 0.36T_{c}$
Galileo E1C	CBOC −	4	-	$\pm 0.34T_{c}$
Galielo E6B	BPSK(5)	1	1	$\pm 0.2T_{c}$
Galielo E6C	BPSK(5)	1	-	$\pm 0.2T_{c}$
Galileo E5b-I	BPSK(10)	1	4	$\pm 0.1T_{c}$
Galileo E5a-I	BPSK(10)	1	20	$\pm 0.1T_{c}$
Galileo E5a/B-Q	BPSK(10)	1	-	$\pm 0.1T_{c}$
Galileo E5	AltBOC(15,10)	1	4 (E5b-I)	$\pm 0.017T_{c}$
Meta-signal E5b-E6BC	AltBOC(35,10,5)	1	1 (E6B)	$\pm 0.00725T_{c}$
Meta-signal E5a-E6BC	AltBOC(50,10,5)	1	1 (E6B)	$\pm 0.005T_{c}$

**Table 2:** Galileo E5 and Meta-signals Time-delay Estimation Standard Deviation (cm). Results for Long Integration Times and $C/N_{0}=45$ dB-Hz.

SNR${}_{\mathrm{out}}$ (dB)	$T_{I}$	AltBOC(15,10)	AltBOC(35,10,5)	AltBOC(50,10,5)
25	10 ms	8	3.55	2.51
30	30 ms	4.47	2	1.41
35	100 ms	2.5	1.12	0.79
40	300 ms	1.41	0.63	0.45
45	1 s	0.8	0.35	0.25
50	3 s	0.45	0.2	0.14

**Table 3:** Phase and Time-delay Estimation Standard Deviation for: GPS L1 C/A ($F_{s}=10$ MHz), L5 ($F_{s}=10$ MHz), Galileo E5 ($F_{s}=120$ MHz), E5b-E6 ($F_{s}=240$ MHz), and E5a-E6 ($F_{s}=400$ MHz).

SNR${}_{\mathrm{out}}$ (dB)	$T_{I}$	$\hat{\varphi }\left(\hat{\tau }\right)$	$\hat{\tau }$ L1 C/A	$\hat{\tau }$ L5	$\hat{\tau }$ E5	$\hat{\tau }$ E5b-E6	$\hat{\tau }$ E5a-E6
15	1 ms	5 mm	7.08 m	2.03 m	-	-	-
18	2 ms	3.6 mm	5.01 m	1.44 m	18 cm	-	-
21	4 ms	2.5 mm	3.55 m	1.02 m	13 cm	5.62 cm	3.94 cm
25	10 ms	1.6 mm	2.24 m	64 cm	8 cm	3.55 cm	2.51 cm
28	20 ms	1.1 mm	1.59 m	46 cm	6 cm	2.51 cm	1.76 cm

## Appendix A. Joint Time-Delay and Phase Estimation Cramér–Rao Bound

Recall that the observation model of interest in Equation (1) is

(A1) $$x=\alpha c\left(\tau \right)+n,\;x,n\in C^{N},\;\alpha \in C,n∼\mathrm{CN}\left(0,\sigma_{n}^{2}I_{N}\right),$$

which can be reparameterized as

(A2) $$x=\rho c^{\prime }\left(\theta \right)+n,\;c^{\prime }\left(\theta \right)=c\left(\tau \right)e^{j\varphi },\;\rho \in R^{+},\theta^{⊤}=\left(\varphi ,\tau \right),$$

or using a real and imaginary part representation, ∀A∈CQ×R,A_=ReAImA,

(A3) $$\underline{x}=\rho {\underline{c}}^{\prime }\left(\theta \right)+\underline{n},\;\underline{n}∼N\left(0,\frac{\sigma_{n}^{2}}{2}I_{2N}\right),$$

where the unknown deterministic parameters to be estimated are $\epsilon^{⊤}=\left(\sigma_{n}^{2},\rho ,\theta^{⊤}\right)$. The corresponding CRB is given by the Slepian–Bangs Formula [39],

(A4) $$\begin{array}{c} \underline{x}∼N\left(m_{\underline{x}}\left(\epsilon \right),C_{\underline{x}}\left(\epsilon \right)\right)\Rightarrow {\mathrm{CRB}}_{\epsilon }=F_{\epsilon }^{-1},\\ {\left(F_{\epsilon }\right)}_{k,l}={\frac{\partial m_{\underline{x}}\left(\epsilon \right)}{\partial \epsilon_{k}}}^{T}C_{\underline{x}}^{-1}\left(\epsilon \right)\frac{\partial m_{\underline{x}}\left(\epsilon \right)}{\partial \epsilon_{l}}+\frac{1}{2}tr\left(C_{\underline{x}}^{-1}\left(\epsilon \right)\frac{\partial C_{\underline{x}}\left(\epsilon \right)}{\partial \epsilon_{k}}C_{\underline{x}}^{-1}\left(\epsilon \right)\frac{\partial C_{\underline{x}}\left(\epsilon \right)}{\partial \epsilon_{l}}\right). \end{array}$$

For the sake of simplicity in the following equations, we note ${\underline{c}}^{\prime }≜{\underline{c}}^{\prime }\left(\theta \right)$, $c≜c\left(\tau \right)$. Considering the model in Equation (A3), $m_{\underline{x}}\left(\epsilon \right)=\rho {\underline{c}}^{\prime }\left(\theta \right)$ and $C_{\underline{x}}\left(\epsilon \right)=\frac{\sigma_{n}^{2}}{2}I_{2N}$, then

(A5) $${\mathrm{CRB}}_{\epsilon }=\left[\begin{array}{cc} \frac{\sigma_{n}^{4}}{N} & 0^{T}\\ 0 & {\mathrm{CRB}}_{\left(\rho ,\theta \right)} \end{array}\right],\;{\mathrm{CRB}}_{\left(\rho ,\theta \right)}=\frac{\sigma_{n}^{2}}{2}{\left[\begin{array}{cc} {∥{\underline{c}}^{\prime }∥}^{2} & \rho {\underline{c}}^{\prime T}\frac{\partial {\underline{c}}^{\prime }}{\partial \theta^{T}}\\ \rho {\frac{\partial {\underline{c}}^{\prime }}{\partial \theta^{T}}}^{T}{\underline{c}}^{\prime } & \rho^{2}{\frac{\partial {\underline{c}}^{\prime }}{\partial \theta^{T}}}^{T}\frac{\partial {\underline{c}}^{\prime }}{\partial \theta^{T}} \end{array}\right]}^{-1}.$$

Using the block matrix inversion lemma ([40]),

(A6) $${\left[\begin{array}{cc} A_{11} & A_{12}\\ A_{21} & A_{22} \end{array}\right]}^{-1}=\left[\begin{array}{cc} C_{1}^{-1} & -A_{11}^{-1}A_{12}C_{2}^{-1}\\ -C_{2}^{-1}A_{21}A_{11}^{-1} & C_{2}^{-1} \end{array}\right],$$

where $C_{1}=A_{11}-A_{12}A_{22}^{-1}A_{21}$, $C_{2}=A_{22}-A_{21}A_{11}^{-1}A_{12}$ and $C_{1}^{-1}=A_{11}^{-1}+A_{11}^{-1}A_{12}C_{2}^{-1}A_{21}A_{11}^{-1}$, then

$${\mathrm{CRB}}_{\left(\rho ,\theta \right)}=\left[\begin{array}{cc} {\mathrm{CRB}}_{\rho } & {\mathrm{CRB}}_{\rho ,\theta }\\ {\mathrm{CRB}}_{\rho ,\theta }^{T} & {\mathrm{CRB}}_{\theta } \end{array}\right],$$

with

(A7) $${\mathrm{CRB}}_{\rho }=\frac{\sigma_{n}^{2}}{2}{\left({\underline{c}}^{\prime T}\Pi_{\frac{\partial {\underline{c}}^{\prime }}{\partial \theta }}^{⊥}{\underline{c}}^{\prime }\right)}^{-1},\;{\mathrm{CRB}}_{\theta }=\frac{\sigma_{n}^{2}}{2\rho^{2}}\Phi_{\theta }^{-1},\;\Phi_{\theta }^{-1}={\left(\frac{\partial {\underline{c}}^{\prime }}{\partial \theta^{T}}\right)}^{T}\Pi_{{\underline{c}}^{\prime }}^{⊥}\frac{\partial {\underline{c}}^{\prime }}{\partial \theta^{T}}.$$

Using the following equality

A_TB_=ReATReB+ImATImB=ReAHB,

we have that

c_′Tc_′=cHc∂c_′∂θTT∂c_′∂θT=Re−je−jφcHe−jφ∂c∂τHjejφcejφ∂c∂τ=Rec2−jcH∂c∂τj∂c∂τHc∂c∂τH∂c∂τ∂c_′∂θTT∂c_′∂θT=c2ImcH∂c∂τImcH∂c∂τRe∂c∂τH∂c∂τ,c_′T∂c_′∂θT=Ree−jφcHjejφcejφ∂c∂τ=0RecH∂c∂τ,

which lead to

(A8) Φθ=∂c_′∂θTT∂c_′∂θT−1c_′Tc_′c_′T∂c_′∂θTTc_′T∂c_′∂θT=c2ImcH∂c∂τImcH∂c∂τRe∂c∂τH∂c∂τ−RecH∂c∂τ2c2.

Finally, using again Equation (A6),

$$\Phi_{\theta }^{-1}=\left[\begin{array}{cc} C_{1}^{-1} & -A_{11}^{-1}A_{12}C_{2}^{-1}\\ -C_{2}^{-1}A_{21}A_{11}^{-1} & C_{2}^{-1} \end{array}\right]$$

C2=Re∂c∂τH∂c∂τ−RecH∂c∂τ2c2−ImcH∂c∂τ2c2=∂c∂τH∂c∂τ−cH∂c∂τHcH∂c∂τc2=∂c∂τHΠc⊥∂c∂τC1−1=1c2+ImcH∂c∂τ2C2c4,−C2−1A21A11−1=−ImcH∂c∂τC2c2,

which allow us to express the CRB as

(A9) $$\begin{array}{ccc} {\mathrm{CRB}}_{\theta } & = & \left[\begin{array}{cc} {\mathrm{CRB}}_{\varphi } & {\mathrm{CRB}}_{\tau ,\varphi }\\ {\mathrm{CRB}}_{\tau ,\varphi } & {\mathrm{CRB}}_{\tau } \end{array}\right], \end{array}$$

(A10) $$\begin{array}{ccc} {\mathrm{CRB}}_{\tau } & = & \frac{\sigma_{n}^{2}}{2\rho^{2}}\frac{1}{{\frac{\partial c\left(\tau \right)}{\partial \tau }}^{H}\Pi_{c\left(\tau \right)}^{⊥}\frac{\partial c\left(\tau \right)}{\partial \tau^{T}}}, \end{array}$$

(A11) CRBφ=σn22ρ21cτ2+CRBτImcτH∂cτ∂τ2cτ4,

(A12) CRBτ,φ=−CRBτImcτH∂cτ∂τcτ2.

Using the relation in Equation (A7),

(A13) CRBρ=σn22cHc−0RecH∂c∂τTRe∂c∂τH∂c∂τ−ImcH∂c∂τ2cHc−10RecH∂c∂τT−1=σn22cHc−Re∂c∂τH∂c∂τ2Re∂c∂τH∂c∂τ−ImcH∂c∂τ2cHc−1=σn221cHc1+RecH∂c∂τ2cHc∂c∂τHΠc⊥∂c∂τ=σn22cτ2+ρ2CRBτRecτH∂cτ∂τ2cτ4.

Moreover, from [16],

$$\begin{array}{ccc} \lim_{\min\left(N_{1},N_{2}\right)\to \infty }{∥c\left(\tau \right)∥}^{2} & = & F_{s}w_{1}=F_{s}\left(\frac{1}{F_{s}}c^{H}c\right)=c^{H}c,\\ \lim_{\min\left(N_{1},N_{2}\right)\to \infty }{∥\frac{\partial c\left(\tau \right)}{\partial \tau }∥}^{2} & = & F_{s}w_{2}=F_{s}\left(F_{s}c^{H}\mathrm{Vc}\right)=F_{s}^{2}c^{H}\mathrm{Vc},\\ \lim_{\min\left(N_{1},N_{2}\right)\to \infty }c{\left(\tau \right)}^{H}\frac{\partial c\left(\tau \right)}{\partial \tau } & = & F_{s}w_{3}=F_{s}\left(c^{H}\Lambda c\right)=F_{s}c^{H}\Lambda c, \end{array}$$

and we finally obtain the CRB from the signal samples as

(A14) $$\begin{array}{ccc} {\mathrm{CRB}}_{\theta } & = & \left[\begin{array}{cc} {\mathrm{CRB}}_{\varphi } & {\mathrm{CRB}}_{\tau ,\varphi }\\ {\mathrm{CRB}}_{\tau ,\varphi } & {\mathrm{CRB}}_{\tau } \end{array}\right], \end{array}$$

(A15) CRBτ=12SNRout1Fs2cHVccHc−ImcHΛccHc2,

(A16) CRBφ=12SNRout1+ImcHΛccHc2cHVccHc−ImcHΛccHc2,

(A17) CRBτ,φ=12SNRoutImcHΛccTcFscHVccHc−ImcHΛccHc2,

(A18) $$\begin{array}{ccc} {\mathrm{CRB}}_{\rho } & = & \frac{\sigma_{n}^{2}}{2c^{H}c}. \end{array}$$
